# Rationale and design of a multi-center, open-label, randomised clinical trial comparing HIV incidence and contraceptive benefits in women using three commonly-used contraceptive methods (the ECHO study)

**DOI:** 10.12688/gatesopenres.12775.2

**Published:** 2018-03-13

**Authors:** G. Justus Hofmeyr, Charles S. Morrison, Jared M. Baeten, Tsungai Chipato, Deborah Donnell, Peter Gichangi, Nelly Mugo, Kavita Nanda, Helen Rees, Petrus Steyn, Douglas Taylor, ECHO Trial Team

**Affiliations:** 1 Effective Care Research Unit, Universities of Witwatersrand and Fort Hare, Eastern Cape Department of Health, East London, South Africa; 2 Global Health, Population and Nutrition, FHI 360, Durham, NC, USA; 3 Departments of Global Health, Medicine, and Epidemiology, University of Washington, Seattle, WA, USA; 4 Department of Obstetrics and Gynaecology, University of Zimbabwe, Harare, Zimbabwe; 5 Vaccine and Infectious Disease Division, Fred Hutchinson Cancer Research Center, Seattle, WA, USA; 6 University of Nairobi, Nairobi, Kenya; 7 Ghent University, Ghent, Belgium; 8 International Centre for Reproductive Health (ICRH), Mombasa, Kenya; 9 Kenya Medical Research Institute, Nairobi, Kenya; 10 Department of Global Health, University of Washington, Seattle, WA, USA; 11 Wits RHI, University of the Witwatersrand, Johannesburg, South Africa; 12 World Health Organization, Geneva, Switzerland

**Keywords:** contraception, HIV acquisition, effectiveness, randomized trial, DMPA, IUD, implants

## Abstract

**Background:** *In vitro*, animal, biological and observational clinical studies suggest that some hormonal methods, particularly depot medroxyprogesterone acetate – DMPA, may increase women’s risk of HIV acquisition. DMPA is the most common contraceptive used in many countries worst affected by the HIV epidemic. To provide robust evidence for contraceptive decision-making among women, clinicians and planners, we are conducting the Evidence for Contraceptive Options and HIV Outcomes (ECHO) study in four countries with high HIV incidence and DMPA use: Kenya, South Africa, Swaziland, and Zambia (Clinical Trials.gov identifier NCT02550067).

**Study design: **We randomized HIV negative, sexually active women 16-35 years old requesting effective contraception and agreeing to participate to either DMPA, the copper T 380A intrauterine device or levonorgestrel implant. Participants attend a contraception support visit after 1 month and quarterly visits thereafter for up to 18 months. Participants receive a standard HIV prevention package and contraceptive side-effect management at each visit. The primary outcome is HIV seroconversion. Secondary outcomes include pregnancy, serious adverse events and method discontinuation. The sample size of 7800 women provides 80% power to detect a 50% relative increase in HIV risk between any of the three method pairs, assuming 250 incident infections per comparison.

**Ethical considerations: **Several WHO consultations have concluded that current evidence on HIV risk associated with DMPA is inconclusive and that a randomized trial is needed to guide policy, counselling and choice. Previous studies suggest that women without a specific contraceptive preference are willing to accept randomization to different contraceptive methods. Stringent performance standards are monitored by an independent data and safety monitoring board approximately every 6 months. The study has been conducted with extensive stakeholder engagement.

**Conclusions: **The ECHO study is designed to provide robust evidence on the relative risks (HIV acquisition) and benefits (pregnancy prevention) between three effective contraceptive methods.

## Introduction

Women living in sub Saharan Africa (SSA) face an unacceptably high risk of maternal mortality, with an estimated mortality ratio of >500 per 100,000 live births ^1, 2^. In addition, these women are at substantial risk of HIV infection. Every week 7000 adolescent girls and young women in eastern and southern Africa become HIV-infected, and adolescents remain the only group in which deaths due to AIDS are not decreasing ^3, 4^. Progestogen only contraception, including the injectables intramuscular depot medroxyprogesterone acetate (DMPA IM), subcutaneous (SC) DMPA, and norethisterone enanthate (NET-EN), as well as levonorgestrel and etonogestrel implants, are used by >60 million women worldwide ^3^ and substantially reduce risk of maternal morbidity and mortality. However, an increasing number of in vitro, animal, biological and observational clinical studies have raised the possibility that hormonal contraception (particularly DMPA IM) may increase a woman’s risk of HIV acquisition. Several recent meta-analyses have found 40–50% increased risks of HIV acquisition among women using DMPA IM compared to women not using hormonal contraception ^5, 6^; sparse data are available for other methods including implants and IUDs. In response, the World Health Organization (WHO) recently changed its guidance for women at high risk of HIV infection using injectable progestogens from a medical eligibility criteria (MEC) category 1 (the contraceptive method can be used without restriction) to a MEC category 2 (the advantages of using the contraceptive method generally outweigh the theoretical or proven risks) ^7^. The guidance stated that “There continues to be evidence of a possible increased risk of acquiring HIV among progestogen-only injectable users. Uncertainty exists about whether reports of any possible increased risk are due to methodological issues with the evidence or a real biological effect.” ^7^. Providing robust evidence to address the uncertainty surrounding this issue is of profound importance to public health programs, to contain the HIV epidemic among women and ensure that women have access to safe and effective contraception to prevent maternal and infant morbidity and mortality.

### The ECHO Consortium

The gold standard for evaluation of a clinical intervention is a randomized clinical trial (RCT) and results from a well-conducted RCT would permit clear guidance for policymakers and programs, clearly formulated counselling messages, and ultimately allow women to make informed choices. The possibility of an RCT comparing effective contraceptive methods and HIV acquisition risk has been raised since the 1990’s. However, questions surrounding evidence (sufficient to motivate a trial, insufficient to make a trial unnecessary), logistics (whether possible to randomize participants), ethics (to randomize vs. providing a choice of contraception, and/or to provide a method that may increase HIV risk), and funding stalled efforts to initiate such a study. Programs remained uncertain about how to counsel women in these settings, given the limitations and inconsistency in the evidence. In response, the ECHO Consortium was founded in 2012 as representatives of FHI 360, the University of Washington, and the University of the Witwatersrand Reproductive Health and HIV Institute (Wits RHI) came together with The Bill & Melinda Gates Foundation to plan a randomized trial of effective contraception (initially including DMPA, NET-EN, the levonorgestrel implant and the copper IUD) and HIV acquisition. In 2013, the WHO joined the leadership of the Consortium to address the concern that a planned 2-arm WHO trial might prejudice the possibility of comparing DMPA with a variety of other effective contraceptive methods. In December 2015, with considerable external stakeholder input, we launched the Evidence for Contraceptive Options and HIV Outcomes (ECHO) Trial.

### Study protocol

The ECHO study protocol is registered at Clinical Trials.gov (Identifier NCT02550067) and with the WHO as part of their clinical trials database. The complete protocol is available as a Supplementary File (Protocol v5.0, revised 3 March 2017). The ECHO Trial completed enrollment on 12 September 2017, with completion of participant follow up expected during the second half of 2018 and publication of results in early 2019.

### Study objectives

The ECHO trial objectives are:

Primary objective: To answer the public health question of the relative risks (HIV acquisition) and benefits (pregnancy prevention) of three commonly-used, effective contraceptive methods (DMPA IM, LNG implant, and the copper IUD) among women in high risk HIV settings who desire effective contraception;

Secondary objectives: To compare pregnancy rates, rates of adverse events that are serious or lead to method discontinuation, and contraceptive method discontinuation rates among the three study methods;

Tertiary objectives: To evaluate whether a) age and b) HSV-2 infection modify the hormonal contraception and HIV acquisition relationship; to evaluate the effect of contraception on early HIV disease progression among seroconverters.

## Methods

### Study outcomes

ECHO study outcomes include:

Primary study endpoint: HIV infection as measured by documented HIV seroconversion (defined by the study HIV algorithm) occurring post-enrolment (see Supplementary materials, Appendix 7);

Secondary endpoints: Pregnancy, method-related serious adverse events, method related adverse events resulting in method discontinuation, and method discontinuation;

Tertiary endpoints: include a) HIV infection by age (<25 years versus ≥25 years) and b) by HSV-2 status; and c) HIV plasma viral load and CD4 count.

### Questions the ECHO trial will and will not address

For HIV-negative women in a setting with high HIV risk who desire effective contraception, the ECHO study will provide robust evidence on the relative benefits and risks of the study methods on important outcomes such as HIV acquisition, pregnancy, method discontinuation, and side effects. Additionally, the study will address whether age and HSV-2 status modify the hormonal contraception and HIV acquisition relationship, as previous data regarding these possible modifying factors are conflicting ^8– 17^. Finally, the study will provide robust data about whether the three methods influence HIV disease progression.

However, the ECHO study will not provide information on the absolute effect of contraceptive methods on HIV risk (compared with no contraceptive use). It is also not powered to detect smaller effects than provided for by the sample size calculation (see ‘Study power and effect size’ below). The ECHO study will also not provide information on the risks of contraceptive methods not included in the study, such as NET-EN, DMPA SC, etonogestrel (ENG) implants, the levonorgestrel IUD, or estrogen containing methods such as combined oral contraceptives (OCs), injectables, patches, or rings.

### Study design

The ECHO Trial is a multi-centre, open-label, randomised clinical trial designed to compare the benefits and risks, including HIV acquisition, between women randomized to one of three commonly used, effective contraceptive methods.

### Randomization

We used a 1:1:1 random allocation method (master randomization list generated using SAS, SAS Inc., Cary NC) and assigned allocation using a predetermined sequence, concealed from all study staff prior to randomisation. The study is open-label due to the difficulty of blinding either clinicians or study participants to the contraceptive arm. However, all study leadership (except for an unblinded study statistician) are blinded to the study outcome by contraceptive group.

### Study metrics

To do the ECHO trial well, the team, funders, and data and safety monitoring board (DSMB) agreed prior to initiation that key operational metrics ( Table 1) would be reviewed continuously by the DSMB during the study and if not met would trigger careful reevaluation of whether to continue the trial:

**Table 1.** ECHO trial operational metrics.

ECHO Performance Standard	Target
#1 Accrual	Achieve target sample within **~**18 months
#2 Method refusal	<5% of subjects *
#3 Retention	Per-visit completion of ≥90% and ≤10% of expected person-years lost *
#4 Method discontinuation	≤10% of all person-time off assigned method *
#5 HIV incidence	sufficient to meet the study objectives (≥3.5%/year)
#6 Ineligible enrolments	<1–2% of total *
#7 HIV endpoint reporting	up-to-date for each DSMB review *
#8 Data quality	current for each DSMB meeting, QC≤5/100 CRFs, ** fax time ≤7d **

* = overall, at each site, in each arm ** QC = quality control, CRFs = case report forms

### Study setting

The study includes women from settings with high HIV incidence and high use of hormonal contraception (particularly DMPA IM) in four countries (South Africa, Kenya, Zambia and Swaziland) across eastern and southern Africa.

### Study population

We enrolled sexually active, HIV-negative women, 16–35 years old, seeking effective contraception, willing to be randomised to any of the three study arms and not desiring pregnancy for the 18 months of study participation. Women were recruited from family planning/reproductive health clinics, clinics serving post-partum and post-abortion clients, other relevant clinics, and the general community.

### Inclusion and exclusion criteria

Complete inclusion and exclusion criteria can be found in the study protocol that is provided as a Supplementary File. Briefly, key inclusion and exclusion criteria include:

Inclusion criteria

- 16–35 years of age (previously pregnant 16 and 17 years where permissible by national regulations and local IRB approval)
- HIV-seronegative
- Wants to use effective contraception
- Agrees to be randomised to either DMPA, LNG implant, or copper IUD
- Agrees to use assigned method for 18 months
- If has had a recent third trimester birth, is at least 6 weeks postpartum at time of enrolment
- Is sexually active (has had vaginal sex within the last 3 months) or was pregnant within the last 3 months

Exclusion criteria

- Medical contraindications (Category 3 or 4 criteria as detailed in the WHO MEC ^1^ to DMPA, LNG implant, or copper IUDs, including
  - Untreated mucopurulent cervicitis on examination, untreated pelvic inflammatory disease (PID), or untreated known gonorrhoea or chlamydia. The participants may be enrolled after treatment.
- Has received a DMPA or NET-EN injection in the last 6 months
- Has used an implant or an IUD in the last 6 months
- Is pregnant or intending to become pregnant in the next 18 months
- Has had a hysterectomy or sterilization
- Has any condition (social or medical), which in the opinion of the investigator, would make study participation unsafe or complicate data interpretation.

### Trial contraceptive methods

DMPA IM 150 mg/1 ml (Depo Provera, Pfizer) is a 3-monthly progestogen-only injectable with a 0.2% failure rate with perfect use, but a 6% failure rate with typical use ^18^. The LNG implant (Jadelle, Bayer) consists of two silicone rods each containing 75 mg of LNG and is highly effective and user independent, with failure rates of <1% for both perfect and typical use ^19^. The T-380A copper IUD, when inserted correctly, has failure rates of <1% in the first year, and only 2.2% in the first 10 years of use ^20^. The contraceptive methods were purchased or donated by USAID or the South African government. The manufacturers were not involved in the design or execution of the trial.

### Visit schedule

Participants are seen at screening, enrolment and at 1, 3, 6, 9, 12, 15 and 18 months. At screening, study staff conducted administrative and regulatory procedures (including obtaining written informed consent for screening), provided contraceptive and HIV counselling, tested for chlamydial and gonococcal infections, did pelvic examinations, obtained reproductive health data and tested for HIV using parallel rapid tests. Sites scheduled women for enrolment visits within 1–42 days of screening. At enrolment, staff obtained informed consent for enrolment, women were randomly allocated to and received their study contraceptive method, received risk reduction and contraceptive counselling, a limited behavioural and clinical assessment, and were tested for pregnancy. Women are treated at the enrolment visit if positive STI results have been received by that time. Otherwise, participants are called to return to the study site for treatment as soon as possible after the results are received.

At 1-month, participants were seen to address any initial side effects, receive further counselling on their contraceptive methods, review relevant adverse events, and confirm IUD and implant presence. Subsequent follow-up visits at 3, 6, 9, 12, and 15 months consist of contraceptive counselling, limited behavioural assessment, review of relevant adverse events, syndromic assessment/treatment of reproductive tract infections (RTIs), provision of male and/or female condoms, assessment for pregnancy, and provision of injectable contraception, as appropriate. At each visit, study staff counsel women on HIV risk reduction and collect blood for HIV rapid testing and, at 6 months only, for plasma archiving. The trial anticipated that novel prevention interventions, such as pre-exposure prophylaxis (PrEP), would become available and recommended during the study period, and the trial protocol encourages counseling about these interventions and access through provision or referral to local centers with appropriate expertise. At the final study visit women received a pelvic examination, and endocervical swabs for gonococcal and chlamydial testing and archiving, urine hCG, and blood for plasma archiving were collected. If HIV seroconversion is suspected at any visit, sites proceed with a physical examination, confirmatory testing (Western Blot and/or HIV EIA, with HIV RNA PCR) and CD4 testing.

### Statistical analyses

The primary analysis will include computation of the hazard ratios of HIV seroconversion based on a proportional hazards (PH) regression model, stratified on site. In the primary analysis, participants will be analysed according to their randomised contraceptive method, regardless of method switching; only participants who are found to have been HIV-infected at enrolment or who fail to contribute a follow-up HIV test result will be excluded. Two pre-planned, supportive analyses of the primary objective will be conducted: a Perfect-use analysis and an As-used analysis. These analyses may incorporate inverse probability of treatment (IPT) and/or inverse probability of censoring (IPC) weights in an effort to account for potential time-dependent confounding and/or informative censoring mechanisms. The results of these supportive analyses will be used to assist interpretation of the primary findings (e.g., to identify caveats regarding the presence or absence of treatment effects with respect to possible causal mechanisms of action). Additionally, analysis plans for secondary and tertiary objectives can be found in the study protocol ( Supplementary File 1) in sections 8.2.2 and 8.2.3.

### Ethical statement

Ethical approval was obtained from the Protection of Human Subjects Committee (PHSC) of FHI 360 (approval number: 523201-146) and from the Ethics Committee (EC) of the WHO (approval numbers: A65897 and A65922). Each participating site also obtained approval from appropriate local Institutional Review Boards.

### Study interim monitoring and DSMB

An independent DSMB convenes approximately every six months to review and evaluate the accumulated study data for participant safety, study conduct and progress, and HIV acquisition risk, and make recommendations to the study team concerning the continuation, modification, or termination of the trial.

## Discussion

### Key decisions in the ECHO study rationale and design

Failure to undertake this RCT would leave a critical public health question unanswered. If the data suggesting harm are true, and programs continue to offer injectable progestogens to HIV negative women in high HIV incidence settings without evidence-based reservations, then the HIV epidemic will have a powerful on-going driver. A recent model concluded that if injectable contraceptive use increased the risk of HIV infection by 1.2–2.19-fold, it could result in 27,000–130,000 new infections per year globally; 87–88% of these additional infections would occur in Southern and Eastern Africa ^21^. Conversely, if false concerns about increased HIV risk persuade policymakers to discourage use or restrict provision of injectable progestogens, then stopping use could cause at least 18,000 more maternal deaths per year globally, and likely even greater maternal morbidity ^21^. Additionally, spillover of restrictions to injectable progestogens to settings with low HIV incidence and high DMPA use would be even more harmful. If the message continues to be confusing, then health care workers may stop providing injectables, even in settings with low HIV burden, and women may be scared away from an effective, relatively safe, inexpensive, widely available and accepted contraceptive method. Answering this question is thus critical for family planning policies, for HIV prevention, and for the health of women.

### Choice of study population

We chose to enrol and study 16–35-year-old women from South and Eastern Africa at high risk of HIV infection and who desired effective contraception because this is the population most affected by a possible association between hormonal contraceptive use and HIV acquisition. We sought sites in diverse East and Southern African countries so that the trial results would be broadly generalizable to women in East and Southern Africa.

### Choice of interventions

The study was designed to provide information on the comparative (HIV) risks and (pregnancy prevention) benefits of three effective contraceptive methods. HIV risks have not been clearly established for any of the three methods and each could plausibly have multiple (and contradictory) effects on HIV risk. A placebo-controlled trial was not believed either ethical or realistic as a placebo provides no contraceptive protection.

We included DMPA because it is the contraceptive that observational data suggest has the highest potential HIV risk and is the most prevalent method in SSA. Use of long acting reversible methods, such as implants, are rapidly increasing in SSA, with sparse data on HIV risk, so implants were an important method to include. We chose the 5-year 2-rod LNG implant above the ENG implant because it is more widely used in Africa overall, and LNG is the progestin used most widely in other contraceptives (e.g. OCs), and also being tested in new multi-purpose technologies that prevent both pregnancy and HIV. Additionally, some data suggest that LNG may be less immunosuppressive than ENG ^22^.

We included the copper IUD to have a highly effective non-hormonal comparator. The copper IUD (380A) is approved for 10 years of use, is registered widely in Africa and is one of the most effective and cost-efficient reversible contraceptives available. The copper IUD is not regarded as an inactive ‘placebo’ because its effect with respect to HIV acquisition is unknown.

We considered and eventually eliminated alternative contraceptives for the following reasons:

- Combined oral contraceptives (OCs): Although estrogen may mitigate potential effects of progestogens on HIV risk ^23, 24^, and COCs are widely used in many African settings, daily adherence to COCs is both poor and difficult to measure. High discontinuation and pregnancy rates could bias study results. Furthermore, estrogen containing methods may increase some health risks and have more contraindications to use, thus limiting the study population.
- Combined injectable: The combined injectable contains estrogen and is not registered or used in most of SSA. Additionally, it is shorter acting (1-month) and may have higher discontinuation and failure rates.
- Condom-only arm: Condoms are not highly effective contraceptives in typical use and their use is partner dependent and thus it is unethical to randomize women seeking highly effective contraception to this method. Moreover, all study participants were counseled to use condoms, which would have made the implementation of a condom use arm problematic.
- NET-EN: As with DMPA, the two-month injectable NET-EN is acceptable to many women in part because injectables are a ‘hidden’ method and convenient to use. If DMPA is found in the trial to have higher HIV risk, it may be important to have an alternative injectable that women and family planning providers could turn to as an acceptable substitute. Limited data suggests that NET-EN might be associated with lower HIV risk than DMPA, but the methods are combined in WHO recommendations as a Category 2, while implants remain a Category 1. Though often used in South Africa, NET-EN is not widely available in other African regions. When financial constraints limited the trial to three study arms, we decided to include a potentially lower risk hormonal method (i.e. an implant), rather than NET-EN.
- The 3-year 1-rod ENG implant received the bulk of the South Africa implant tender, and has the advantage of a pre-loaded insertion device. However, the 5-year 2-rod LNG implant was chosen for reasons explained above.

### Study power and effect size

Before finalization of the study protocol, the Bill & Melinda Gates Foundation supported formative research to assess the level of increased HIV risk associated with a contraceptive that would be meaningful from a policy and programmatic perspective. Interviews were conducted with African MOH officials, clinicians, and epidemiologists. The consensus was that any proposed study should be able to detect at least a 50% increased HIV risk associated with one contraceptive relative to another. The ECHO Trial was thus designed to have 80% power to detect a 50% increase in risk of HIV acquisition among women randomised to different contraceptive methods. Due to an *a priori* expectation of method switching, we accounted for a dilution of effect from a “true” 50% to an “apparent” 45% increase in HIV risk. The type I error was chosen to control the family-wise error rate for the three primary comparisons at 0.10: each of the three individual comparisons will be assessed with a two-sided type I error rate of 0.04. A desirable property of the 3-arm design is that if only one method has an increased risk of 50% then there is greater than a 90% chance of concluding it is harmful relative to one or both other methods. The total sample size of 7,800 women was selected assuming an underlying HIV incidence of 3.5 per 100 woman-years, up to 18 months of prescribed follow-up per woman, and a maximum of 10% loss to follow-up or early discontinuation.

### Ethical considerations

Randomization to contraceptive methods has historically been controversial. Individual choice is the cornerstone of family planning provision and policy, and a strongly held opinion has been that randomization is incompatible with the prerogative of choice. However, this assumes that all women have only one contraceptive preference. Previous randomized trials ^25, 26^ have shown that many women do not have a clear preference for a specific method, and in the context of a research study with high level counseling and consent, are prepared to agree to randomization between alternative effective methods.

Secondly, an ethical prerequisite for any randomized trial is equipoise regarding the benefits and risks of the alternative interventions. Colleagues have challenged the ethics of the ECHO trial on the grounds that data from biological and observational studies on DMPA and HIV risk are sufficiently persuasive to guide practice and render randomization unethical ^27– 30^. On the other hand, several WHO consultations occurring both prior to and during the ECHO Trial that included exhaustive reviews of the literature and assessment by independent expert panels from various disciplines have concluded that the evidence is inconclusive. Furthermore, any weighing of the relative benefits and risks needs to include all relevant effects, such as the reduction in the risks of unintended pregnancy and the value of DMPA as an effective, acceptable and confidential contraceptive method.

### Study feasibility

Prior to the start of the trial, interested colleagues voiced concerns about the feasibility of the trial including a) the feasibility of enroling and randomizing 7,800 women to different contraceptive methods, b) achieving high contraceptive method continuation in the trial, and c) enroling a study population with sufficient HIV incidence ^27, 28^. Addressing the first concern, the ECHO trial has emphasized enroling only women that are truly willing to using any of the three methods by counseling those who appear to favor one method over another not to enrol. At screening, women receive extensive counseling on all the risks and benefits of each study method; they also leave the study site after screening and return 1 to 42 days later for enrolment. This gives women a chance to reflect on their participation and willingness to use any of the three methods as well as necessitating an additional action (returning to the study site) to enrol in the study. The study metrics agreed upon prior to the study (outlined above) specified an acceptable enrolment rate and rate of refusal to be randomized and plans were in place to stop the trial if enrolment performance was poor. On 12 September 2017 ECHO Trial closed recruitment, having randomized 7,830 women with low refusal rates (data not shown).

The second feasibility issue – achieving high contraceptive method continuation – was a salient concern for the study investigators as many family planning programs have significant rates of discontinuation of the three study methods within the first 12–18 months, and high contraceptive discontinuation would adversely affect trial integrity. The trial has a goal of achieving 90% contraceptive continuation for each of the study methods (i.e. <10% loss of follow-up time on the study method). Accordingly, the ECHO Team has put significant resources into training (and retraining) study clinicians on contraceptive clinical and counseling techniques. Issues affecting contraceptive continuation are discussed on weekly calls and several highly trained consortium staff members are available on a daily basis to respond to contraceptive issues arising at sites. In addition, the study included a 1-month follow-up visit to specifically address side effects and other concerns with the contraceptive methods.

The feasibility of ECHO was also questioned in relation to accruing sufficient HIV endpoints to have adequate study power. The trial is endpoint driven, defined by the target of observing at least 250 incident HIV infections per pairwise comparison. Based on enrolment of 7800 women, follow-up of 18 months, and a maximum 10% loss to follow-up or early method discontinuation, the trial requires an underlying HIV incidence of 3.5/100 person-years. A site selection committee researched and visited more than 30 possible sites using multiple selection criteria including HIV incidence (≥ 3.0/100 person-years per site and an overall incidence across all study sites of ≥ 3.5/100 person-years). We used multiple data sources to estimate incidence rates for sites (or their surrounding areas) including data from recently completed HIV prevention trials (e.g., FACTS, ASPIRE, VOICE, etc.) to select a group of sites projected to have sufficiently high HIV incidence.

Finally, several scientific colleagues argued that the funding for an RCT of DMPA and HIV acquisition, even if ethical and feasible, could be better spent on other areas of research and programming such as expanding the contraceptive method mix in East and Southern Africa ^27– 29^. Such arguments suggest that investments in programming and research are a zero-sum game and that money invested in the ECHO trial would necessarily take funding away from programmes aimed at increasing access to a variety of effective contraceptive methods. Additionally, these arguments assume that other methods such as implants and IUDs are not associated with HIV risk. However, the ECHO team believes that there are sufficient funds to simultaneously address increased method mix and provide program managers and women with the highest quality scientific evidence upon which to make their family planning decisions ^31, 32^. Moreover, the ECHO trial, by training large numbers of clinicians and counselors and providing large numbers of women with these highly effective methods in four East and Southern African countries, serves as a catalyst for subsequent large scale provision of these methods ^32^. Finally, the trial will provide much needed information on the relative risk of copper IUDs and LNG implants on HIV risk relative to DMPA.

## Conclusions

Young women in parts of sub-Saharan Africa continue to have high incidence of HIV infection as well as high morbidity and mortality from unintended pregnancy. It is unclear whether DMPA plays a role in increased HIV susceptibility. The ECHO Trial has been designed to provide the highest quality evidence to resolve this important public health question. Thorough attention has been given to various design characteristics such as the choice of study arms, the effect size that it is designed to detect, and the ethical and feasibility challenges. Some of the challenges, such as the feasibility to enrol and randomize women to the three study arms have now been successfully mitigated. Others, including high method continuation and retention, continue to receive great attention. We anticipate that the ECHO Trial will provide high quality evidence on the risks and benefits of the three contraceptive methods and will thus allow women, clinicians, program managers and policymakers to make informed decisions about contraceptive choices for women at high risk of HIV infection.

## Notes

^1^World Health Organization (WHO), *Medical eligibility criteria for contraceptive use*. 5th ed 2015, Geneva: WHO

## Supplementary material

Supplementary File 1: ECHO Trial Protocol (version 5.0, revised 3 March 2017).
