# Supplementary material for: Rationale and design of a multi-center, open-label, randomised clinical trial comparing HIV incidence and contraceptive benefits in women using three commonly-used contraceptive methods (the ECHO study)
Source: Gates Open Res. 2018 Mar 13;1:17. Originally published 2017 Dec 29. [Version 2] doi: 10.12688/gatesopenres.12775.2 (PMC5771152; doi:10.12688/gatesopenres.12775.2)
Supplement: Peer review history

# Peer review: Referee response for version 1

**DOI:** 10.21956/gatesopenres.13835.r26178

**Published:** 2018-01-15

**Authors:** Sten Vermund

**Affiliations:** 1 Yale School of Public Health, Yale University, New Haven, CT, USA

The ECHO trial team has produced a highly useful protocol synopsis, along with an ethical rationale for the study itself.  This reviewer found it clear and highly informative.

1. P.3: This sentence is helpful: “The ECHO Trial has currently completed enrollment and is following up study participants.”  However, a timeline for deliverables should be provided, as this will be every reader’s first question! Also, the word “currently” won’t play well in the article since readers will read this in 2024 as well as in 2018, so an anchor date should be substituted, e.g., “As of October 1, 2017, the ECHO Trial had completed enrollment…. (or some such).”
2. Table 1: Can the authors comment how operational metrics are were met in the first year, say, of the trial’s conduct?  This could be brief, or even via an additional column in the Table.
3. P.5 (statistical analysis):  This section was exceedingly brief.  Given that there are 3 arms, that both intent-to-treat and as-treated analyses are proposed, and that the as-treated analysis will not follow randomization, a bit more analytic detail is warranted.
4. P.6 (choice of interventions): While the IUD impact on HIV acquisition is unknown, I think that the authors should mention that its safety for use in HIV-infected women is established with RCT-level evidence (PMID: 17689627).  This is important as a rationale for women who seroconvert in the trial to continue IUD use, if they wish. 
5. P.6 (sample size):  It seems that powering the study to a 47% difference between groups is the study’s principal limitation.  Somewhere in the discussion, the prospect that a false negative trial will result if the incidence difference is a 1/3 ^rd^ or 2/5 ^ths^, rather than a full 47%.

Additional note: Since this is a paper about trial methods, the "datasets" question is not really applicable.

I have read this submission. I believe that I have an appropriate level of expertise to confirm that it is of an acceptable scientific standard.

# Response

**Published:** 2018-02-26

**Authors:** Charles Morrison

**Affiliations:** FHI 360, USA

The ECHO trial team has produced a highly useful protocol synopsis, along with an ethical rationale for the study itself.  This reviewer found it clear and highly informative.

1. P.3: This sentence is helpful: “The ECHO Trial has currently completed enrollment and is following up study participants.”  However, a timeline for deliverables should be provided, as this will be every reader’s first question! Also, the word “currently” won’t play well in the article since readers will read this in 2024 as well as in 2018, so an anchor date should be substituted, e.g., “As of October 1, 2017, the ECHO Trial had completed enrollment…. (or some such).”

Author response:  We have edited the paper as follows: “The ECHO Trial completed enrollment on 12 September 2017, with completion of participant follow up expected in 2018 and publication of results in 2019.”  And under study feasibility: “On 12 September 2017 ECHO Trial closed recruitment, having randomized 7,830 women with low refusal rates (data not shown).”

 

1. Table 1: Can the authors comment how operational metrics are were met in the first year, say, of the trial’s conduct?  This could be brief, or even via an additional column in the Table.

Author response: As this is an ongoing randomized trial, our policy is to restrict disclosure of post-randomization data (other than reporting in confidence to the DSMB).  Thus, we reported on the number of women enrolled and randomized but no other operational metrics.

 

1. P.5 (statistical analysis):  This section was exceedingly brief.  Given that there are 3 arms, that both intent-to-treat and as-treated analyses are proposed, and that the as-treated analysis will not follow randomization, a bit more analytic detail is warranted.

Because this manuscript focuses on the rationale for the study design and because we include the full study protocol as supplemental material, we have purposefully kept this section short.  Nevertheless, we have added several additional sentences describing planned perfect-use and as-used analyses.  Additional description can be found in section 8.2 of the study protocol.

 

1. P.6 (choice of interventions): While the IUD impact on HIV acquisition is unknown, I think that the authors should mention that its safety for use in HIV-infected women is established with RCT-level evidence (PMID: 17689627).  This is important as a rationale for women who seroconvert in the trial to continue IUD use, if they wish. 

Author response:  Yes, there is limited data suggesting that the copper IUD is safe for HIV-infected women and WHO’s medical eligibility criteria for contraceptive use gives this a ‘2’ rating – i.e. that the benefits of use generally outweigh the risks for IUD use for women with asymptomatic or mild HIV disease.  However, we would also need to then discuss the issue of DMPA and Lng implant use among HIV infected women.  Because this issue was not salient to our choice of contraceptives to include in the study, it does not seem necessary or important to include this in this manuscript.

1. P.6 (sample size):  It seems that powering the study to a 47% difference between groups is the study’s principal limitation.  Somewhere in the discussion, the prospect that a false negative trial will result if the incidence difference is a 1/3 ^rd^ or 2/5 ^ths^, rather than a full 47%.

Author response:  First, this was a mistake, the manuscript was changed to reflect the correct amount (45% difference).  Secondly, we added under ‘Questions the ECHO Study will address’ the following: “It is also not powered to detect smaller effects than provided for by the sample size calculation (see ‘Study power and effect size’ below).”

Additional note: Since this is a paper about trial methods, the "datasets" question is not really applicable.

We agree.

# Peer review: Referee response for version 1

**DOI:** 10.21956/gatesopenres.13835.r26174

**Published:** 2018-01-12

**Authors:** Audrey Pettifor

**Affiliations:** 1 University of North Carolina at Chapel Hill, Chapel Hill, NC, USA

Minor Comments

 What does the * in Table 1 refer to?

 What was the rationale for excluding women who had used DMPA, implant or IUD in the last 6 months?

 Statistical analyses:

 Please describe how the analysis for modification by HSV-2 and age will be conducted. I see this is in the protocol but would be nice to say something brief in the paper.

 Also, describe the analytic plans for examining the effect of contraceptive method on HIV disease progression and is there expected to be power to conduct this analysis? Again I see this in the protocol but would be nice to include something brief or at least say these analyses are described in the protocol?

 How will differential contraceptive method continuation be handled in secondary analyses? I did not see this addressed in the protocol. Given that it is much easier to ‘miss’ a DMPA injection than to have an IUD removed one might hypothesize that it is much more likely there will be lower adherence to the DMPA arm than the IUD arm.

 DSMB- is the DSMB evaluating the study operational metrics in Table 1 to determine study continuation as well? If so might be useful to directly state this.

 Page 7, the authors state that 7,830 women have been recruited and randomized “now”,  consider putting a date (Month/year) of this enrolment number as will be more interpretable. Also, please consider including a possible time line for results of the trial? Late 2019?

 The authors describe the key considerations for this trial well and lay out the key concerns raised about such a trial and why it was still important to conduct.

 Discussion- did the study team come up with a level of risk at which trial would be stopped? In the protocol it says  “To that end, it is anticipated that a method group may be discontinued from further study if it is associated with a significant increased risk of HIV (compared to either other method) when controlling the type I error rate at the two - sided 0.0 4 level using appropriate stopping boundaries.”  Is there any actual increased risk level or is just any increased risk that would stop the method?

 According to the protocol GC/CT are tested for at the screening visit using NAAT. At what visit it treatment provided if positive? Please include this in the paper.

 Would also be good to mention that cervical cancer screening is provided (realize site specific?).

 Also would be good to include the HSV-2 testing information and at what visits given that will be used to examine modification.

I have read this submission. I believe that I have an appropriate level of expertise to confirm that it is of an acceptable scientific standard.
